# Leveraging transfer learning with deep learning for crime prediction

**DOI:** 10.1371/journal.pone.0296486

**Published:** 2024-04-17

**Authors:** Umair Muneer Butt, Sukumar Letchmunan, Fadratul Hafinaz Hassan, Tieng Wei Koh

**Affiliations:** 1 School of Computer Sciences, Universiti Sains Malaysia, Penang, Malaysia; 2 Department of Computer Science, The University of Chenab, Gujrat, Pakistan; 3 Department of Computer and Information Sciences, Universiti Teknologi Petronas, Seri Iskandar, Perak; Sunway University, MALAYSIA

## Abstract

Crime remains a crucial concern regarding ensuring a safe and secure environment for the public. Numerous efforts have been made to predict crime, emphasizing the importance of employing deep learning approaches for precise predictions. However, sufficient crime data and resources for training state-of-the-art deep learning-based crime prediction systems pose a challenge. To address this issue, this study adopts the transfer learning paradigm. Moreover, this study fine-tunes state-of-the-art statistical and deep learning methods, including Simple Moving Averages (SMA), Weighted Moving Averages (WMA), Exponential Moving Averages (EMA), Long Short Term Memory (LSTM), Bi-directional Long Short Term Memory (BiLSTMs), and Convolutional Neural Networks and Long Short Term Memory (CNN-LSTM) for crime prediction. Primarily, this study proposed a BiLSTM based transfer learning architecture due to its high accuracy in predicting weekly and monthly crime trends. The transfer learning paradigm leverages the fine-tuned BiLSTM model to transfer crime knowledge from one neighbourhood to another. The proposed method is evaluated on Chicago, New York, and Lahore crime datasets. Experimental results demonstrate the superiority of transfer learning with BiLSTM, achieving low error values and reduced execution time. These prediction results can significantly enhance the efficiency of law enforcement agencies in controlling and preventing crime.

## 1 Introduction

Crime is one of the most intensifying and critical concerns in ensuring the safety and security of the public. Crime has been one of the social issues manipulating the nature of life and economic progress in a community in recent years [1]. The accessibility of modern technology has permissible implementation to gather detailed data on crime [2, 3]. With today’s increasing crime rates, crime analysis is required, including strategies and procedures to reduce the chance of crime [4]. The fundamental component of the sustainable development of a country is security. It is the obligatory duty of a country’s security forces to regulate criminal occurrences and threats to society’s well-being. Governments spend much of their Gross Domestic Product (GDP) on enforcement agencies [5, 6].

**Fig 1:**
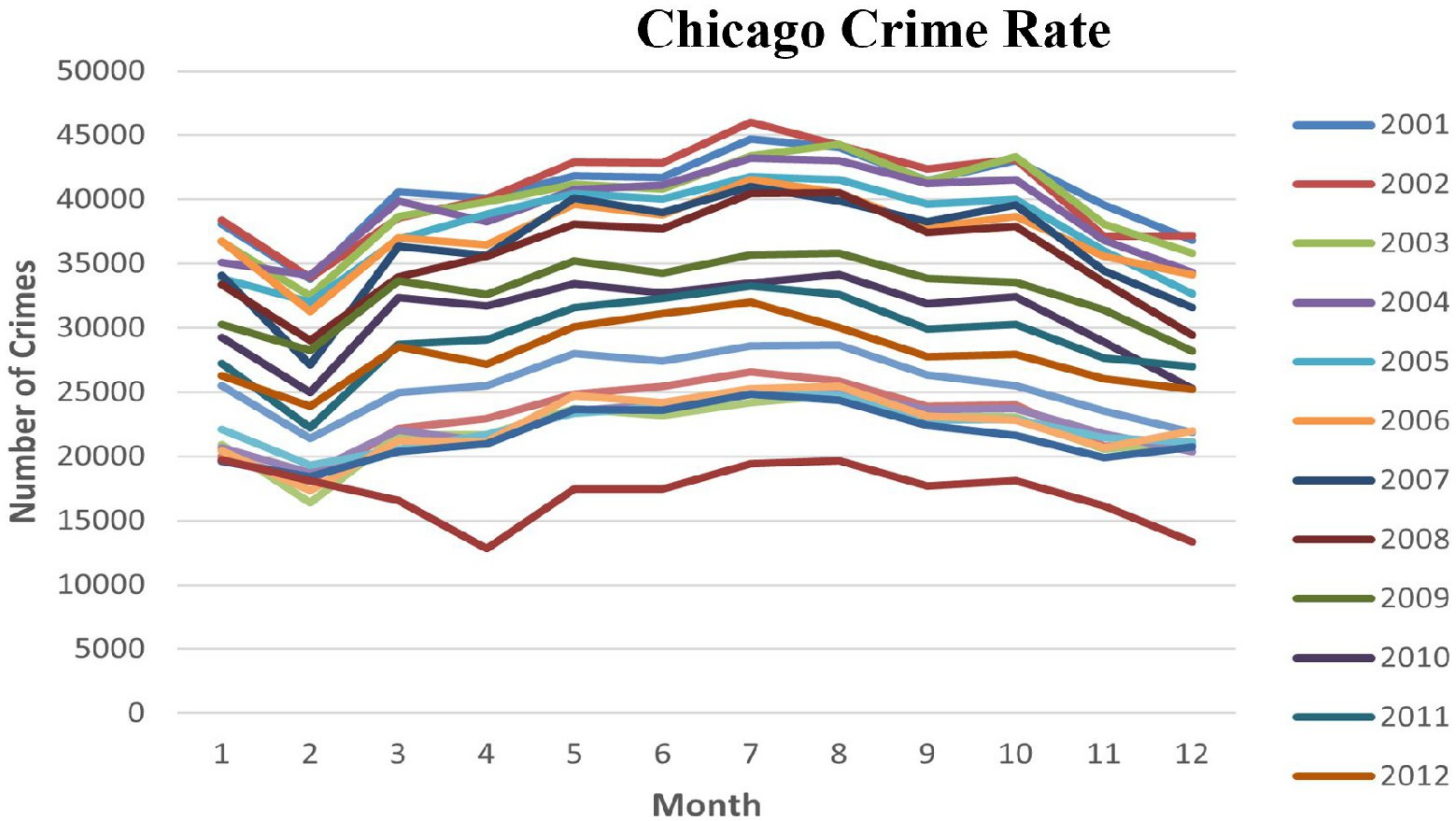
Change in the crime rate of Chicago city from 2001 to 2012.

The priority of law enforcement agencies has been to study crime trends and patterns to formulate an effective policy based on historical data to create a tranquil community [7, 8]. The vast amount of spatiotemporal data has grabbed the attention of scientists in conducting further analyses of criminal interrogation and crime. Depending on past data, crime prediction has been a topic of interest that has gained much attention in analysis, resulting in the proposal of numerous methods by covering multiple aspects associated with crime [9–11]. Crime is frequently seen as a location-specific feature, as some areas pose a more critical threat of criminal activity than others [12]. Fig 1 shows the crime spike variance in Chicago city. It is well known that crime is not distributed evenly, uniformly, or even randomly within a given area, regardless of its size [13]. Spatio-temporal facts within the crime datasets using the Geographic Information System (GIS) have transformed the crime prediction system [14, 15].

Recently, time series analysis strategies such as Autoregressive Integrated Moving Averages (ARIMA) and Seasonal Autoregressive Integrated Moving Averages (SARIMA) produced promising results for crime prediction [16–19] as compared to traditional machine learning techniques. In addition, machine and deep learning methods have been used to predict crime using spatiotemporal data [20, 21]. Moreover, deep learning strategies like CNN and LSTM have additionally remained investigated and shown to be beneficial compared to the cutting-edge approach [22–24]. A hybrid of LSTM and ES gives promising results in predicting financial time series data [25]. Recent literature shows the challenges of forecasting and predicting vicious acts primarily in denser regions of excessive crime through various deep learning and time series analysis models [26, 27].

However, adequate data is necessary to strengthen the crime prediction system [28]. Researchers worldwide study alternative approaches like transfer learning [29, 30] to overcome this issue. In most deep learning models, transfer learning is employed to solve the problem of inadequate data [21]. Ye et al. [31] suggested a unique framework for time series prediction using transfer learning. The primary purpose of this research was to transmit information, or functionality, from the source to the target dataset. However, in some instances, if the targeted dataset is insufficient, the model may be required to learn features or patterns from several source data sets.

Transfer learning has recently been employed in a variety of research domains, such as forecasting traffic [32], predicting financial time series data [33], and forecasting air quality index [34]. For places with similar demographic features and even an exceptional state, it is possible to use transfer learning to predict crime. Transfer learning creates a generic model, incorporating his previous knowledge and performing admirably in the new environment.

This study is divided into three steps. First, the study examines several statistical modelling techniques in finance, economics, and business for time series prediction, such as SMA, WMA, and EMA. Moreover, this study investigates deep learning-based algorithms for time series prediction, such as LSTM, BiLSTMs, and CNN-LSTM algorithms. Finally, a BiLSTM based architecture is proposed by adopting a transfer learning paradigm to overcome the deep learning model’s excessive data availability and training issues. This approach transfers knowledge from one neighbourhood to another, utilizing fewer resources and time.

The rest of the study is as follows: Section 2 discussed state-of-the-art literature on crime prediction and forecasting and used it for transfer learning. Section 3 describes the proposed methodology. Section 4 highlights the significance of the proposed model using experimental evaluation. Next, the performance measures used to conduct the research are presented with an experimental evaluation. Finally, Section 5 concludes the paper by focusing on empirical findings and future directions.

## 2 Related work

This section discusses the two state-of-the-art aspects involved in this research. First, it compares various statistical and deep learning techniques for crime prediction. Second, this study highlights the significance of the transfer learning paradigm in solving massive data availability and model training issues for deep learning and improving the prediction accuracy of various time series problems.

### 2.1 Deep learning and statistical techniques

Several attempts have been reported in the literature on the significance of statistical and deep learning approaches for prediction [17, 27, 35]. Particularly time series analysis techniques such as ARIMA, SARIMA [21], Exponential Smoothing (ES), and Moving averages models [36]. Moreover, Deep learning techniques such as LSTM [37], ST-ResNet [38], and Deep Neural Networks [39] have also been reported for enhancing crime prediction accuracy.

Zhe Li et al. [18] study the inherent traits of Chinese city crime by analyzing crime data from the original case file. First, a quantitative method for case facts is devised, primarily based on Chinese descriptions, which can be utilized to drastically transform the unstructured information within the case record to the model’s safety level. Second, based on the variety of cases, the occurrence of time, and location, assess the core traits of the case. Finally, an ARIMA-based forecasting model is introduced to predict the state of crime over time. Hossain et al. [40] discovered spatiotemporal crime hotspots by examining two distinct real-world crime datasets for Los Angeles (LA) and Denver. The paper demonstrates how the Naive Bayesian and Decision Tree classifiers forecast potential crime types.

Manjunatha and Annappa [41] studied higher crime rates in cities using a predictive method based on spatial analysis and autoregressive models, highlighting the hazardous crime location. For the trial of this approach, two real-world datasets were collected in the cities of New York and Chicago, and the results demonstrate good precision in spatial and temporal crime prediction in each region. Gu and Dai [42] employed time series analysis on meteorological data, health-related data, and economic and stock market indexes.

Mahajan and Mansotra [43] proposed a deep learning-based system to detect cyberbullying on different social network sites. They used the transfer learning concept with deep learning to train a cyber-bullying detection model across Twitter, Wikipedia, and Form Spring. The transfer-based deep learning technique is evaluated on three state-of-the-art real-world datasets. They got an F1 score of 0.94 for Wikipedia and Twitter and 0.95 for the Form Spring dataset. Ying et al. [44] aimed for a CNN-based image retrieval system for crime scene investigation. The suggested technique is based on the feature fusion technique, which exploits transfer learning to extract useful information from crime scenes. Pre-trained models of VGG and PCA are utilized for fine-tuned feature extraction. The proposed algorithm is evaluated on crime scene investigation images provided by Xian University. The algorithm performed comparable to state-of-the-art techniques, with 93.37% precision.

### 2.2 Transfer learning for crime prediction

Recently, transfer learning has been exploited with different classification approaches to address data availability challenges in the real world. Transfer learning aims to get knowledge from one source task and apply it to a target but related task [45]. The study of transfer learning is inspired by the idea that humans can logically use previously acquired knowledge to solve new problems quickly and accurately. Bappee et al. [46] explored transfer learning to predict crime in neighbouring city boroughs. Crime data from New York City from 2012 to 2013 was collected to evaluate the theoretical framework presented in this paper. They identified several research topics that need the serious attention of researchers.

Karl et al. [47] defined transfer learning as follows: Transfer learning for deep neural networks is the process of first training a base network on a source dataset and task and then transferring the learned features (the network’s weights) to a second network to be trained on a target dataset and task. Transfer learning has been widely used in Computer Vision (CV) and Natural Language Processing (NLP). Fuzhen et al. [48] presented an inclusive survey on the significance of transfer learning and its possible usage with existing machine learning algorithms. They discussed the performance of twenty different transfer learning algorithms by evaluating three real-world datasets: the Amazon review, Office No. 31, and Reuters 21578. The experiment’s outcomes showed that transfer learning models should be carefully selected for solving different real-world problems.

Huaxia et al. [49] addressed the data availability problem for certain regions using meta-learning with spatiotemporal prediction. The term “transfer learning” refers to transferring the knowledge of the model learned on sufficient data from a city to other cities where data availability is limited. They investigate the effectiveness of the meta-learning approach with the fusion of transfer learning for spatiotemporal prediction of traffic and water quality in Chicago and Boston cities. Lianbing et al. [50] suggested a hybrid intrusion detection method based on fuzzy C-mean PCA and clustering to overcome different Internet of Things-based security and privacy issues. They exploit transfer learning with the proposed intrusion detection approach for various security factors. The algorithm was assessed on the dataset named KDD-CUP99. Simulations showed a low false-positive value by improving detection accuracy.

Hu et al. [51] suggested a method for forecasting wind velocity for the new farm that involved transferring the information of numerous historical farms. The authors pre-train a two-layer deep neural network model using time-series data from multiple ancient farms. The trained model’s parameters are standard across all wind farms. Therefore, the model may be thought of as a recurring feature transformation. With a model created using multi-source datasets, the overall performance based on a single dataset is not analyzed.

## 3 Proposed methodology

**Fig 2:**
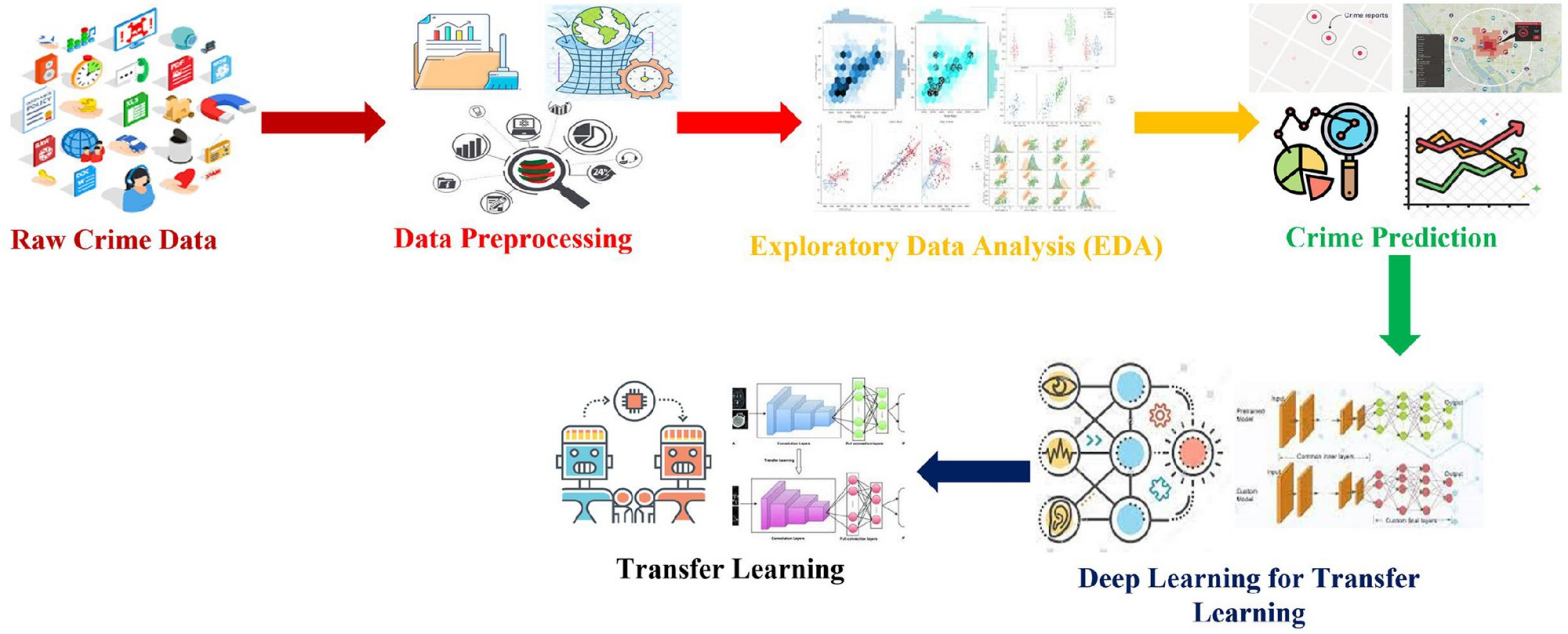
Proposed methodology by leveraging transfer learning for crime prediction.

This section discusses the proposed crime prediction methodology. Moreover, various state-of-the-art prediction methods are fine-tuned, and the best approach is used under the transfer learning paradigm. The methodology comprised several steps required to perform crime prediction using transfer learning, as shown in Fig 2.

### 3.1 Data collection and preprocessing

The dataset utilized in this research comprises criminal data from Chicago, New York, and Lahore. This study obtained publicly accessible datasets from their respective official crime portals, including Chicago [52], New York [53], and Lahore [54]. Common attributes are chosen in each dataset: id, date, time, crime category, crime description, spatial (longitude and latitude), and year. Table 1 shows the data specifications for each city.

**Table 1:** Dataset specification.

Country	Population	Attributes	Records	Categories
**Chicago**	2.7m	22	7.2M	28
**New York**	8.4m	35	2.1M	30
**Lahore**	1.1m	14	1.5M	20

The Chicago city dataset reports crimes from January 2001 to December 2020. The crime dataset of Chicago originally had 7255968 crime records, of which 682341 were eliminated due to incorrect formatting (duplication, values lacking, etc.). The criminal records reported from January 2006 to 2019 are included in the crime dataset of New York, with a population density of 8.4 million in 2019. In the crime dataset of NYC, there were 2158804 records initially, and 45884 records were eliminated during data cleaning. Finally, there have been 2112920 records for experiments in New York. The Punjab Police Department revealed the Lahore City Crime dataset from 2015 to 2016. The crime dataset of Lahore originally had 151638 crime records, of which 18 were eliminated during data preprocessing. Lastly, there have been 151611 records for Lahore for the experiment.

### 3.2 Exploratory data analysis (EDA)

**Fig 3:**
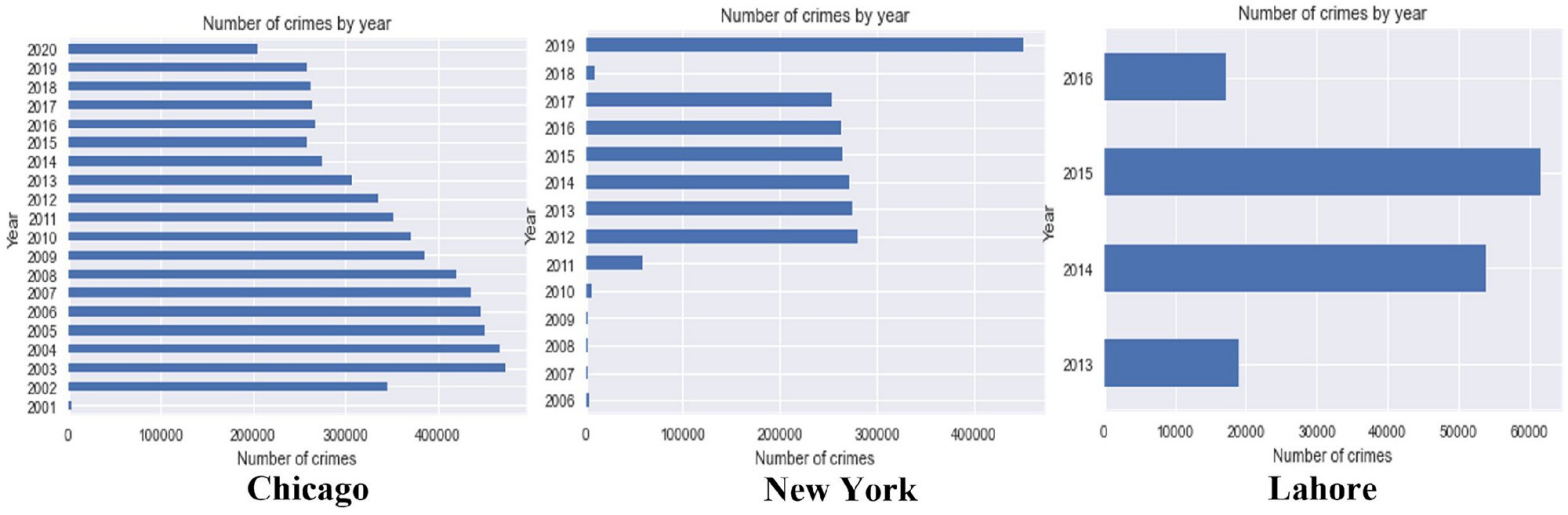
Crime distribution over the years in Chicago, New York, and Lahore.

This section discusses the comprehensive periodic insights of the Chicago, New York, and Lahore datasets. Fig 3 shows crime distribution over the years. Moreover, it shows a decreasing trend of crimes in Chicago and an increasing trend in New York and Lahore. Crimes are reported at the district, borough, and town levels.

The crime datasets reveal that environmental variables like harsh weather or the winter season may reduce crime and favour individuals and residents. It is evident from Table 2 that crime rates were lower in February than in previous months in both Chicago and New York. But in Lahore, June has the lowest crime rate. The highest crime rates were recorded in July in Chicago and January in New York and Lahore. Most crimes were committed Friday in Chicago and New York, while on Thursday in Lahore.

**Table 2:** Comparison of crimes in Chicago, New York, and Lahore based on EDA.

	Datasets		
Comparison	Chicago (2001-2020)	New York (2006-2019)	Lahore (2013-2016)
Crime spike day	Friday	Friday	Thursday
Lowest crime day	Sunday	Sunday	Saturday
Crime spike week	1st week of month	Month’s 1st Week	Month’s 1st Week
Lowest crime week	Month’s 5th Week	Month’s 5th Week	Month’s 5th Week
Crime spike month	Year’s 7th month	Year’s 1st month	Year’s 1st month
Lowest crime month	Year’s 2nd month	Year’s 2nd month	Year’s 6th month
Crime spike date	Month’s 1st day	Month’s 1st day	Month’s 2nd day
Lowest crime date	Month’s last day	Month’s last day	Month’s last day
Crime spike year	2003	2019	2015
Lowest crime year	2001	2007	2013
Crime spike area	District 8	Brooklyn	Iqbal Town
Lowest crime area	District 20	Staten Island	Wagha Town
Top 5 Crimes	Theft, Burglary, Fraud, Narcotics, Assaults	Petit Larceny, Harassment, Assault, Fraud, Theft	Burglary, Auto Theft, Narcotics, Fraud, RushDrive

Table 2 shows the comparison is drawn based on EDA among Chicago, New York, and Lahore crime data. Moreover, Table 2 outlines the top 5 crimes in all regions.

### 3.3 Crime prediction

This section discusses the six state-of-the-art prediction algorithms used for crime prediction. The six most promising statistical and deep learning methods (SMA, WMA, EMA, LSTM, LSTM-CNN, and BiLSTM) are fine-tuned to attain precise predictions on Chicago, New York, and Lahore crime datasets. The following sections explain the methods chosen for experimental investigation.

#### 3.3.1 Statistical methods

In this section, this study discussed the characteristics of each statistical prediction method and highlighted their significance in crime prediction.

*3.3.1.1 Simple Moving Averages (SMV).* An SMA is an arithmetic moving average calculated by adding crime count data (P) for the last few years and dividing the result by the number of years the crime observation occurred (n). It is easy to interpret and efficient, as shown in Eq 1 [55].

$$\begin{array}{c} SMA=\frac{P1+P2+P3+P4+\ldots +P_{x}}{n} \end{array}$$ (1)

*3.3.1.2 Weighted Moving Averages (WMA).* The current data points (P) are given a higher weight (n) than the past data points in this method. This is because the current data points can depict the trend more significantly. The total weightings must equal one (or one hundred percent), as shown in Eq 2. The weighted average is computed by multiplying a particular crime count by its corresponding weighting and adding the results together [55].

$$\begin{array}{c} WMA=\frac{P1*n+P2*\left(n-1\right)+\ldots +P_{n}}{\frac{n*(n+1)}{2}} \end{array}$$ (2)

*3.3.1.3 Exponential Moving Average (EMA)*. An EMA is a moving average (MA) type that gives an exponentially low weight to each previous data point, as shown in Eq 3. An exponentially weighted moving average (EWMA) reacts more strongly to the change in crime trend than a simple SMA, which gives all observations in the period equal weight [55].

$$\begin{array}{c} EMA=(2/n+1)*(close-PreviousEMA)+PreviousEMA \end{array}$$ (3)

#### 3.3.2 Deep learning methods

This section discusses state-of-the-art deep learning methods and compares their performance for crime prediction. In addition, the study utilizes this method later on for transfer learning. The following sections discuss state-of-the-art techniques.

*3.3.2.1 Long Short Term Memory (LSTM).* LSTM-based techniques are an RNN extension that can effectively deal with the vanishing gradient problem. This memory extension can remember information over an extended period, thus allowing interpretation, scripting, and erasing of information from their memories. The memory of LSTM is known as the “gated” cell, where the term gate is enthused by the capability to remember or ignore the memory information [56].

Each memory unit has an input gate, an output gate, a forgetting gate, and a cell status (C, t). These gate architectures let information pass selectively, allowing for the removal or addition of data to the cell state. The preceding sequence of *h*_*t*−1_ controls the contents of the cell state in the prior layer. The sigmoid activation function takes the sequence’s *X*_*t*_ as input to determine whether to keep or discard the top layer of the cell state content, as shown in Eq 4.

$$\begin{array}{c} f_{t}=\sigma \left(W_{f}*\left[h_{t}-1,x_{t}\right]+b_{f}\right) \end{array}$$ (4)

The weights and offsets of each threshold layer are shown in the equation as *W*_*f*_, *b*_*f*_, *W*_*i*_, *b*_*i*_, *W*_*o*_ and *b*_*o*_. The symbol *sigma* denotes the sigmoid activation function. The current sequence position’s input is analyzed, the necessary information is identified, and the cell status is updated. Finally, the input threshold layer and input gates state must be changed, as shown in Eqs 5 and 6.

$$\begin{array}{c} i_{t}=\sigma \left(W_{i}*\left[h_{t}-1,x_{t}\right]+b_{i}\right) \end{array}$$ (5)

$$\begin{array}{c} C_{t}’=tanh(W_{C}*\left[h_{t}-1,x_{t}\right]+b_{C}) \end{array}$$ (6)

This study may update the cell state *C*_*t*1_ to *C*_*t*_ using the forgetting and input gates, as shown in Eq 7. *f*_*t*_ signifies the information selected for deletion, and $i_{t}*C_{t}^{\prime }$ denotes the new information.

$$\begin{array}{c} C_{t}=f_{t}*C_{t}-1+i_{t}*C_{t}’ \end{array}$$ (7)

According to Eq 8, *Ct* is derived from the LSTM unit state, and *C*_*t*_ is updated to *C*_*t*1_ using the input and forgetting thresholds. Finally, the decision of what to output must be made based on the data stored by the cell state or the content of the cell state that has been saved selectively. Like the input gate’s two-part update, the output gate must use the sigmoid activation function to determine the possible output. The output threshold layer *O*_*t*_ will be filtered depending on the unit’s state.

$$\begin{array}{c} O_{t}=\sigma \left(W_{o}*\left[h_{t}-1,x_{t}\right]+b_{o}\right),h_{t}=O_{t}*\left(C_{t}\right) \end{array}$$ (8)

**Fig 4:**
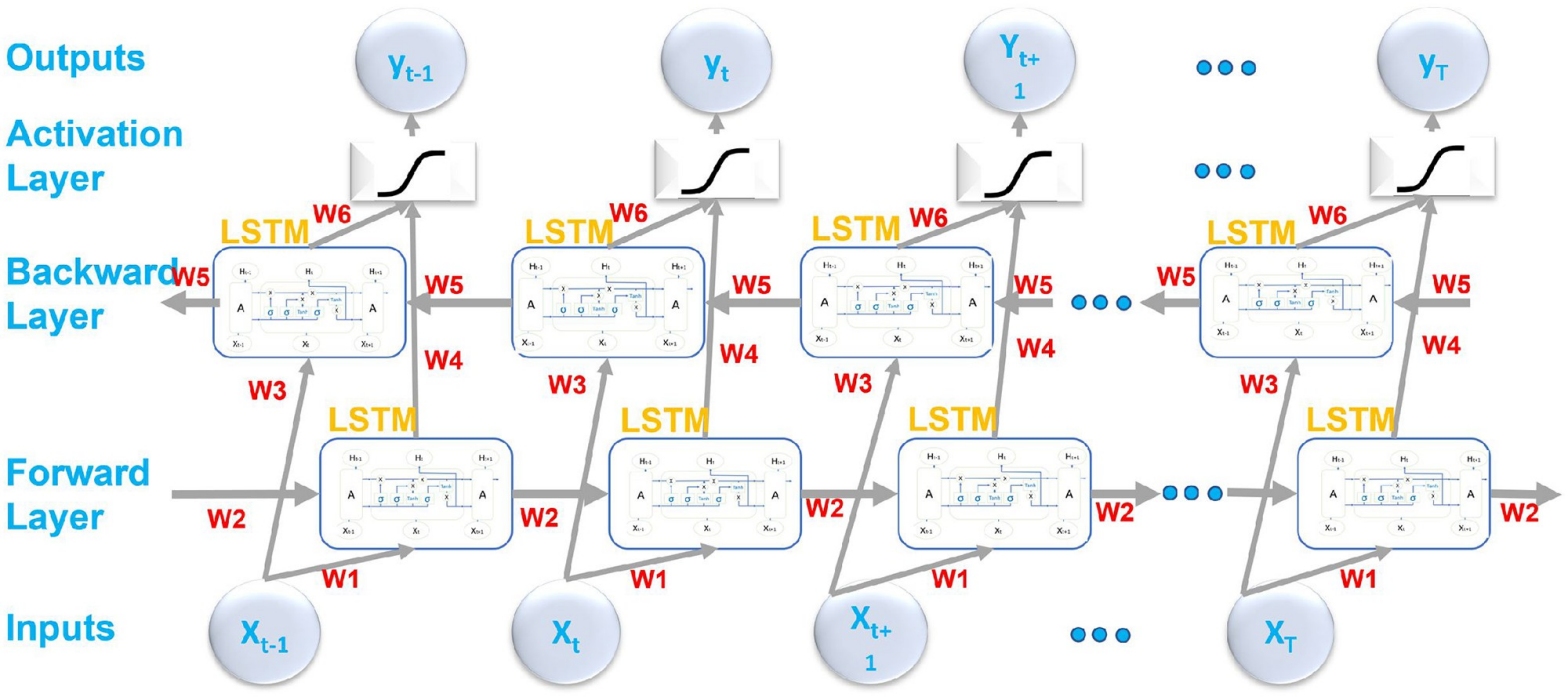
A detailed architecture of BiLSTM for crime prediction.

*3.3.2.2. Bi-directional Long Short Term Memory (BiLSTMs).* There are occasions when it is vital to make the prediction utilizing a lot of past and subsequent information since it is more accurate. Consequently, a two-way cyclic neural network is shown, and Fig 4 shows its construction. The output layer is connected to the forward and backward layers and contains six standard weights, w1–w6. At each time stamp, the six weights are repeated: w1 and w3, which represent input to the forward and backward hidden layers; w2 and w5, which represent information flow from the hidden layers to themselves; and w2, which represents information flow from the forward and backward hidden layers to the output layer (w4 and w6). The enlarged graph is acyclic because there is no information movement between the forward and backward hidden layers [56].

The forward layer obtains and saves the forward calculation from time 1 to time t and the output of the forward hidden layer at each time. The computation is reversed in the backward layer from time t to time 1, and the output of the backward hidden layer at each time is received and preserved. Finally, the final output is created at each instant by merging the findings of the forward and backward layers. The following Eq 9 shows the BiLSTM mathematical expressions.

$$\begin{array}{c} \begin{array}{cc} h_{t} & =f(w1_{x_{t}}+w2h_{t}-1)\\ h_{t}^{\prime } & =f(w3_{x_{t}}+w5_{h_{t}}–1)\\ O_{t} & =g(w4_{h_{t}}+w6_{h_{t}^{\prime }}) \end{array} \end{array}$$ (9)

Algorithm 1 outlines the training and fine-tuning process for BiLSTM. Input to the model are three crime datasets and returned MSE, MAD, and MAE values.

**Algorithm 1** Crime Prediction Using BiLSTM

**Require:** Crime Datasets of Chicago, Newyork, and Lahore

**Ensure:** MAE, MAD, And MSE of predicted data

 ▷ Data Splitting (70% Training and 30% Testing)

1: *size* ⇐ *Length*(*data*) * 0.70

2: *train* ⇐ *data*[0…*size*]

3: *test* ⇐ *data*[*size*…Length(size)]

4: *set random.seed*(8)    ▷Set random seed to an 8 to achieve optimal results

 ▷Fit a BiLSTM model to training data

5: *X* ⇐ *train*

6: *Y* ⇐ *train* − *X*

7: *model* = *Sequential*()

8: *model*.*add*(*Bidirectional*(*LSTM*(*neurons*, *stateful = True*))

9: *model.compile*(*loss* =’ *MSE,MAE,MAE*’, *optimizer* =’ *adam*’)

10: **while** *i* = *range*(*epoch*) **do**

11:  *model*.*fit*(*X*, *y*, *epochs* = 1, *shuffle* = *False*)

12:  *model*.*reset*_*states*()

13: **end while**         ▷Make Predictions

14: *Y*_*predicted*_ ⇐ *model*.*predict*(*Y*)

15: *output* ⇐ *Return*(*MSE*, *MAD*, *MAE*)

*3.3.2.3 Hybrid of Convolution Neural Network and Long Short Term Memory (CNN-LSTM).* A Convolutional Neural Network (CNN) is an artificial neural network with 2D picture input. It automatically extracts and learns features from 1D sequence data, such as univariate time series data, which may be a breeze using CNNs. A convolutional neural network model is frequently employed as part of a hybrid model with a long short-term memory backend for predictions [57]. The convolutional neural network analyzes subsequences of input collectively supplied as a sequence for the long short-term memory model to comprehend. This hybrid model is referred to as Convolutional Neural Networks Short-Term Memory. The first step is to divide the input orders into subsequences that the convolutional neural network model can handle. For example, the study may divide the univariate time series data into input and output samples using four steps and one step as output. Every subsequence of two-time steps may be interpreted by the convolutional neural networks, which can then offer a time series of interpretations to the LSTM model to process as input [58].

## 4 Experimental evaluation

This section evaluates six state-of-the-art statistical and deep learning approaches for crime prediction. Primarily, three state-of-the-art evaluation measures, Mean Absolute Error (MAE), Median Absolute Deviation (MAD), and Mean Squared Error (MSE), are used [27, 59]. Furthermore, three spatiotemporal crime datasets from Chicago [52], New York [53], and Lahore [54] are used for monthly and weekly crime predictions. The following sections discuss the predictions in different experimental settings.

### 4.1 Chicago district-wise prediction for a month and week using statistical and deep learning method

This section illustrates the experimental analysis results to compare the prediction performance of statistical and deep learning methods on Chicago crime data. The crime data for Chicago City is divided into 22 districts. This study split the data into training (70%) and testing (30%) sets to perform monthly and weekly crime predictions for each district of Chicago.

**Fig 5:**
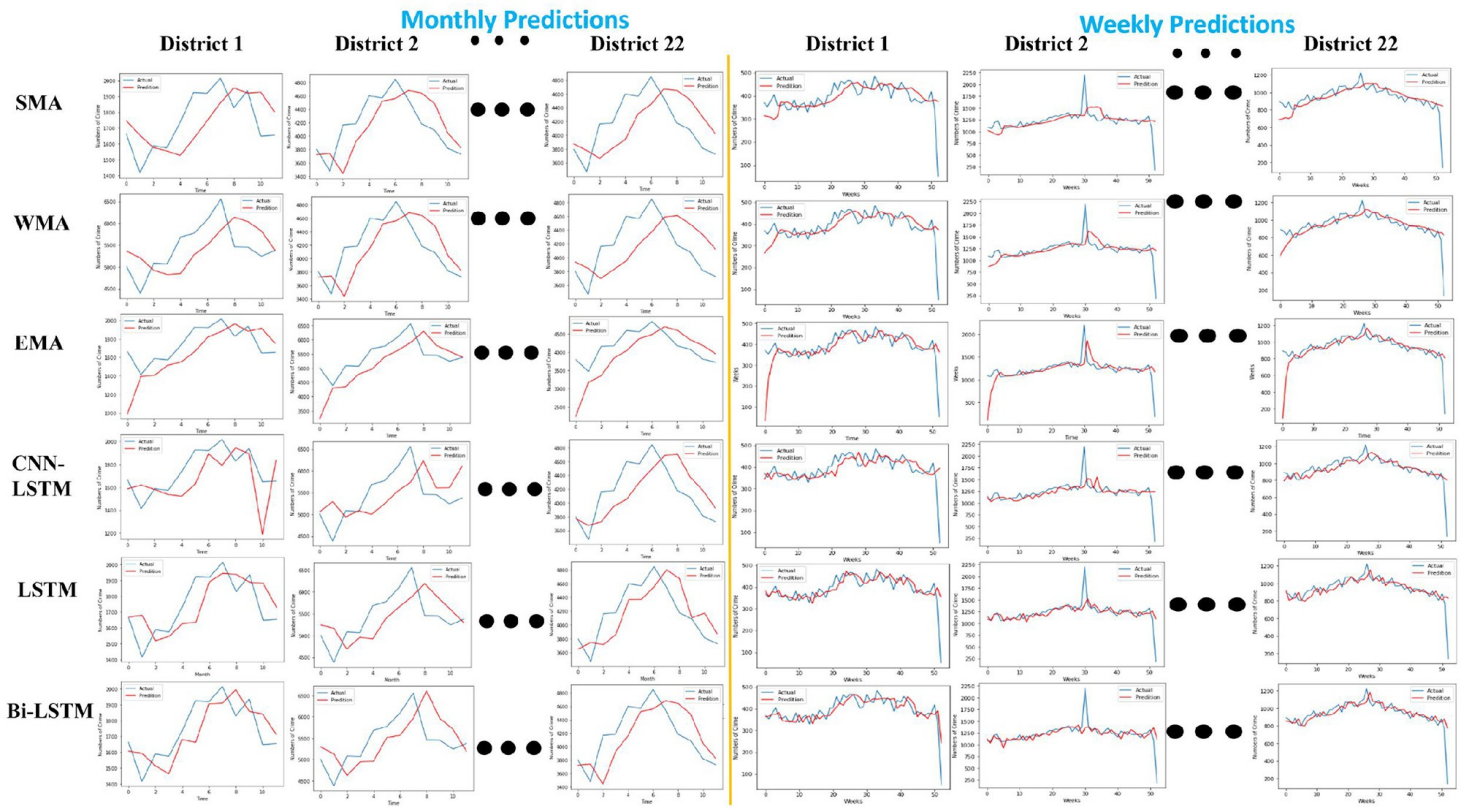
District-wise crime prediction for a month and a week using statistical and deep learning models.

Fig 5 shows the district-wise prediction for a month and a week. The X-axis shows time regarding the number of months, and the crime counts are on the Y-axis. The blue curve is the actual measurement, and the red curve is the prediction measurement of the statistical and deep learning methods. It is evident from Fig 5 and Table 3 that the BiLSTM model performs efficiently with a low error rate compared to other methods. In particular, it achieves a low error rate in weekly predictions compared to monthly predictions.

**Table 3:** Comparison of Chicago district-wise prediction error values for a month and a week using statistical and deep learning methods.

Type	Methods	Monthly			Weekly		
		MAE	MAD	MSE	MAE	MAD	MSE
Statistical Techniques	SMA	840.20	359.65	458.72	76.94	46.10	147.70
	WMA	404.33	388.67	477.99	74.87	42.50	146.94
	EMA	482.76	316.24	672.64	85.66	40.70	188.43
Deep Learning Techniques	CNN-LSTM	333.16	296.71	411.64	68.58	42.99	137.77
	LSTM	332.44	267.03	417.78	63.28	39.49	123.80
	**BiLSTM**	**312.56**	**255.75**	**390.61**	**62.85**	**38.44**	**120.08**

### 4.2 New York borough-wise crime prediction for a month and week using statistical and deep learning methods

**Fig 6:**
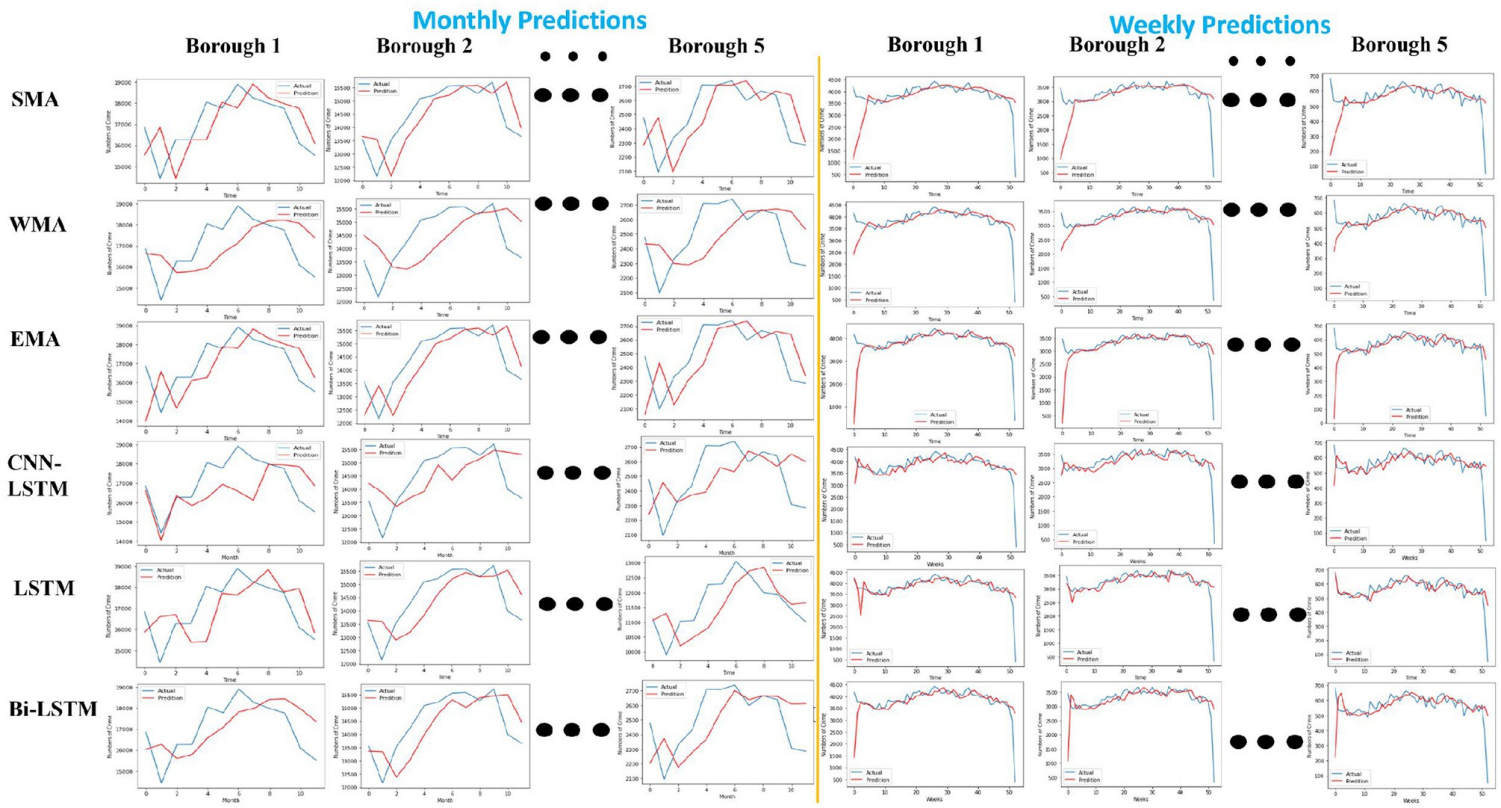
Borough-wise crime prediction for a month and week using statistical and deep learning models.

This section discusses the evaluation results performed on crime data for New York City (NYC), divided into five boroughs (Bronx, Brooklyn, Manhattan, Queens, and Staten Island). This study splits the data into training (70%), and testing (30%) sets randomly to perform monthly and weekly crime predictions for each borough. Fig 6 shows each borough’s monthly and weekly prediction graphs. The X-axis shows time regarding the number of months, and the crime counts on the Y-axis. The blue curve is the actual measurement, and the red curve is the prediction measurement of the statistical and deep learning methods. Table 4 shows the comparative analysis of deep learning and statistical techniques based on MAE, MAD, and MSE values.

**Table 4:** Comparison of Borough-wise prediction error values for a month using statistical and deep learning methods.

Type	Methods	Monthly			Weekly		
		MAE	MAD	MSE	MAE	MAD	MSE
Statistical Techniques	SMA	615.50	413.10	852.82	230.78	79.10	543.35
	WMA	761.95	493.60	982.43	167.23	70.47	399.21
	EMA	696.84	459.72	938.68	189.24	66.92	527.63
Deep Learning Techniques	CNN-LSTM	657.69	592.60	859.04	153.62	77.85	361.28
	LSTM	332.44	267.03	417.78	141.18	68.99	352.20
	**BiLSTM**	**654.74**	**435.12**	**835.51**	**130.59**	**60.29**	**313.34**

The fine-tuned BiLSTM outperformed other deep learning and statistical approaches for monthly and weekly predictions. Moreover, weekly predictions are more accurate with less error rate than monthly predictions.

### 4.3 Lahore town-wise crime prediction for a month and week using statistical and deep learning methods

**Fig 7:**
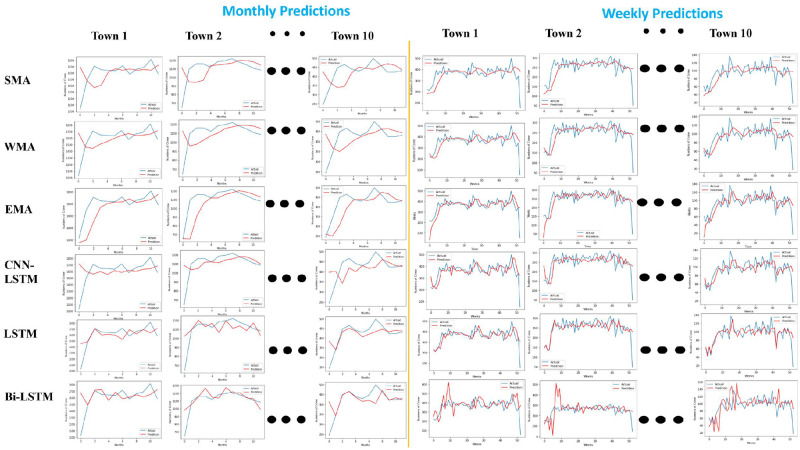
Town-wise crime prediction for a month and a week using statistical and deep learning models.

This section presents the novel spatiotemporal crime dataset of Lahore City, Pakistan. The Lahore dataset is divided into 10 towns: Iqbal Town, Samanabad Town, Gulberg Town, Data Ganj Bakhsh Town, Nishtar Town, Ravi Town, Shalamar Town, Cantonment, Wahga Town, and Aziz Bhatti Town. This study splits data into training (70%) and testing (30%) sets to perform monthly crime predictions. Fig 7 shows the graphs of each town prediction for a month. The X-axis shows time regarding the number of months and the crime counts on the Y-axis. The blue curve is the actual measurement, and the red curve is the prediction measurement of the statistical and deep learning methods.

Fig 7 and Table 5 show the comparative analysis based on MAE, MSE, and MAD for monthly and weekly crime prediction. Again, BiLSTM achieved a low error rate in all towns compared to other statistical and deep-learning models. In addition, the fine-tine BiLSTM achieved a lower error rate in weekly predictions than in monthly predictions. Therefore, BiLSTM has been adopted with the transfer learning paradigm for knowledge transfer.

**Table 5:** Comparison of town-wise prediction error values for a month and a week using statistical and deep learning methods.

Type	Methods	Monthly			Weekly		
		MAE	MAD	MSE	MAE	MAD	MSE
Statistical Techniques	SMA	116.172	73	172.396	27.507	18.3	42.992
	WMA	105.119	65.7	161.96	25.782	17.667	39.957
	EMA	98.25	57.05	154.445	26.832	18.184	41.25
Deep Learning Techniques	CNN-LSTM	88.649	54.028	142.603	27.14	17.805	42.017
	LSTM	66.622	26.399	118.618	20.026	12.405	32.233
	**BiLSTM**	**60.82**	**20.47**	**110.58**	**19.84**	**11.84**	**31.16**

### 4.4 Transfer learning using BiLSTM

**Fig 8:**
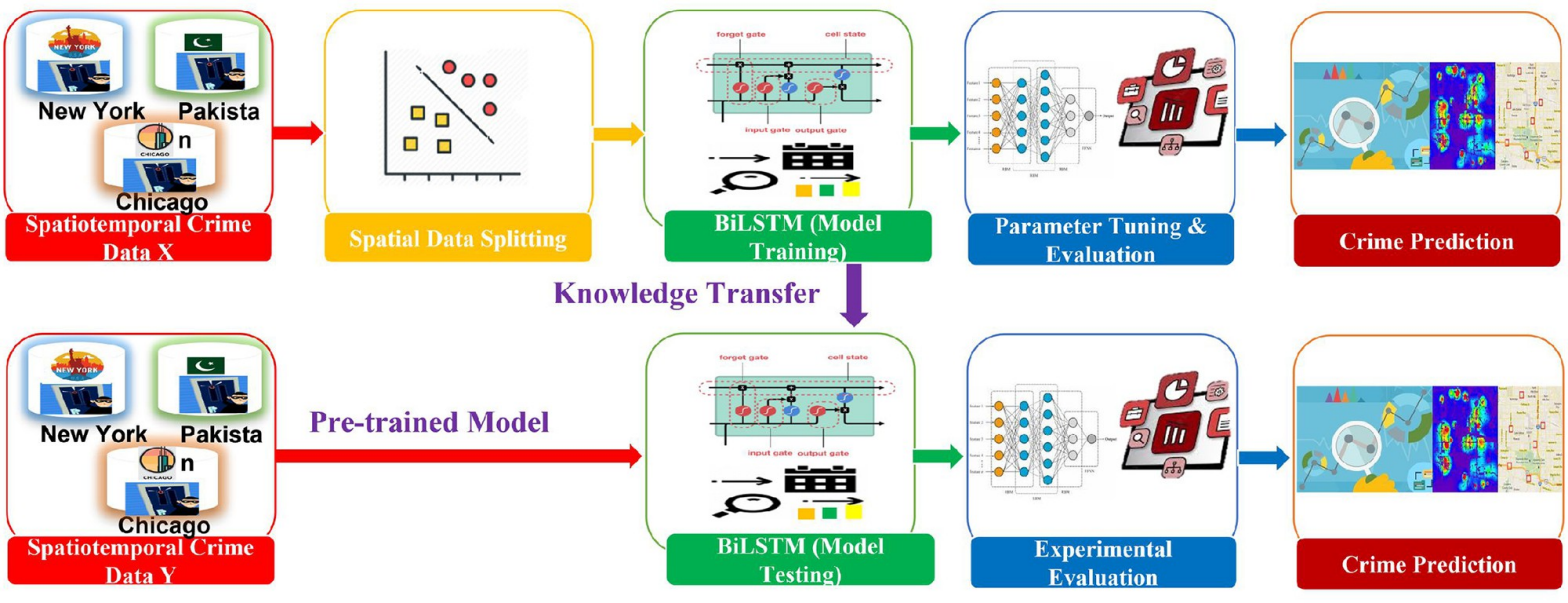
Crime prediction methodology using BiLSTM under transfer learning paradigm.

This section utilizes the BiLSTM under the transfer learning paradigm due to its superior performance in weekly and monthly crime predictions. This study used three spatiotemporal crime datasets from Chicago, New York, and Lahore for experimental evaluation. The proposed BiLSTM based transfer learning methodology is shown in Fig 8. The BiLSTM Based transfer learning on a crime dataset comprises several steps. The first step is to acquire data from the source. Second, the dataset is divided into training and testing subsets. Third, the BiLSTM model is utilized and fine-tuned for crime prediction. Fourth, evaluation is performed using three state-of-the-art crime datasets and evaluation measures. Lastly, transfer learning is achieved between boroughs, districts, and towns.

**Fig 9:**
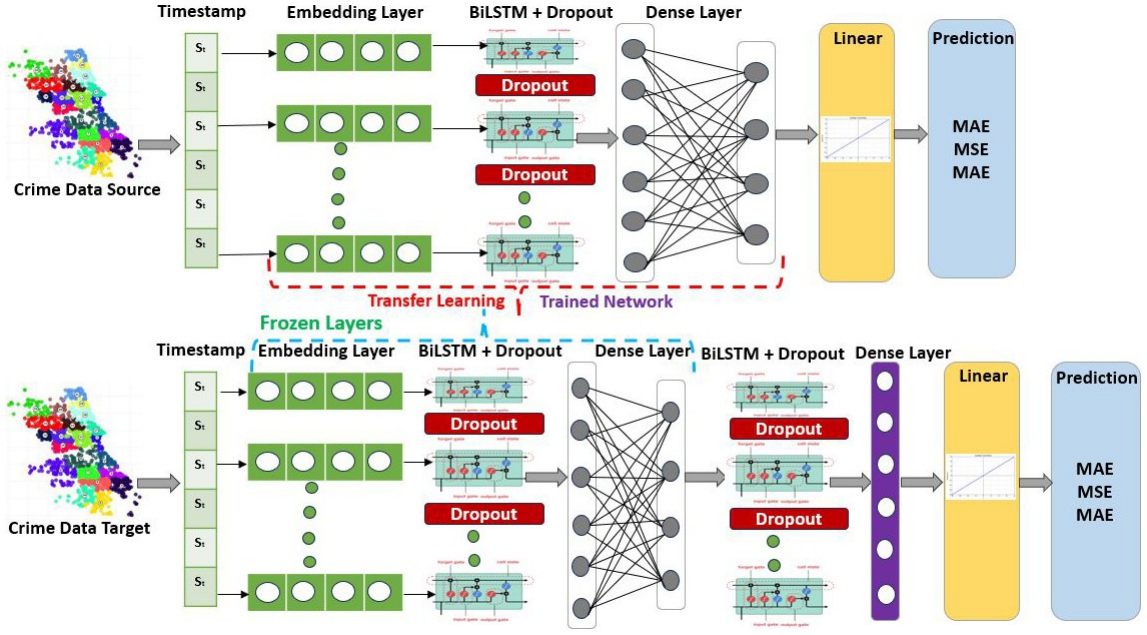
Architecture details of transfer learning using BiLSTM for crime prediction.

Furthermore, the architecture of BiLSTM as a pre-trained model in transfer learning is described in detail in Fig 9. When feeding timestamp data into the model, embedding layers from the crime data extract contextual information. The BiLSTM layer extracts the sequential pattern and semantic data (past and future) from the source data. To avoid model overfitting, dropout layers are also included. Finally, the linear activation function is utilised to reduce the error between the actual and predicted values when dense layers and the linear activation function are applied to extract key features. The following sections discuss the knowledge transfer process in different experimental setups.

#### 4.4.1 Optimization of the proposed approach

**Fig 10:**
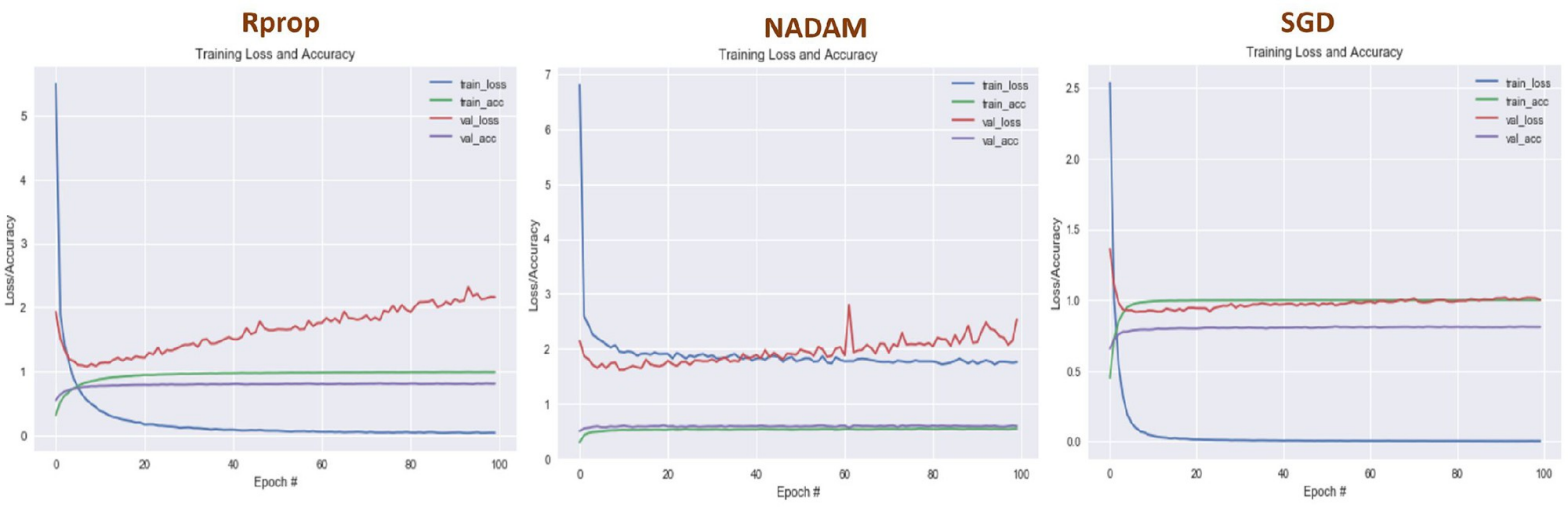
Line graph showing the optimization process using state-of-the-art optimizers for crime prediction.

To fine-tune the design parameters, this study used three cutting-edge optimizers from the Keras library, namely SGD [60], Rprop [61], and Nadam [62]. Once features have been extracted, these optimizers are used to build the model with two dense layers for the final prediction. Rprop achieves the best test accuracy of any, as demonstrated in Fig 10. Rprop optimizer is useful, especially for recurrent neural networks [63, 64]. Based on the gradient’s sign, it modifies the learning rates for each parameter and achieves significant performance compared to others.

#### 4.4.2 Knowledge transfer from a district, borough, and town to another

**Fig 11:**
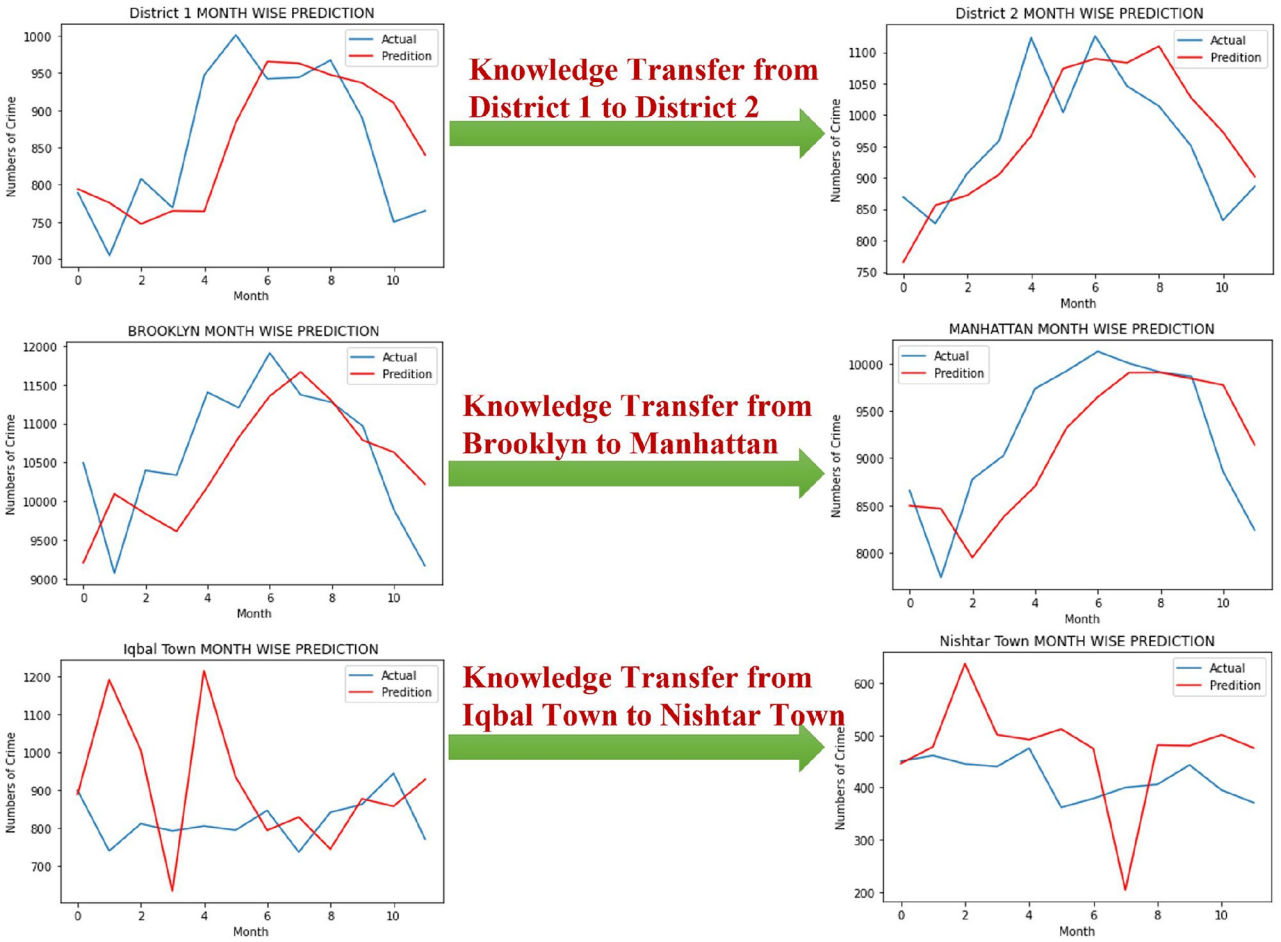
Knowledge transfer from one district1 to district2, Brooklyn to Manhattan, and from Iqbal Town to Nishtar Town.

This section focuses on knowledge transfer from one district of Chicago to another, one borough of New York to another, and from one town of Lahore to another. Primarily from District 1 to District 2 of Chicago, Brooklyn to Manhattan, and Iqbal Town to Nishtar Town, respectively. This study split the data into 70% training and 30% testing to perform monthly crime prediction. The pre-trained model adopts similar parameters and some epochs to test in the target neighbourhood. Fig 11 shows the graphs of a district, borough, and town prediction for the months using transfer learning. The X-axis shows the number of months, and the crime counts are on the Y-axis.

The blue curve is the actual measurement, and the red is the prediction. Table 6 depicts the value of MAE, MAD, MSE, and execution time with transfer learning and without transfer learning using BiLSTM. The monthly crime prediction from pre-trained methods has a constant execution time when tested on the target dataset. It is also observed that the error values and execution time corresponding to monthly crime prediction with transfer learning are significantly less than the results without transfer learning.

**Table 6:** Comparison of error values and execution time of Chicago, New York, and Lahore datasets for monthly crime prediction using BiLSTM.

Datasets	Without Transfer Learning				With Transfer Learning			
	MAE	MAD	MSE	Time	MAE	MAD	MSE	Time
**Chicago**	70.28	63.31	88.43	01:37 Min	65.68	58.66	81.154	00:01 Min
**New York**	734.69	871.96	811.51	01:14 Min	505.93	373.41	621.82	00:01 Min
**Lahore**	155.67	118.78	205.90	00:58 Min	87.53	85.04	107.75	00:01 Min

**Fig 12:**
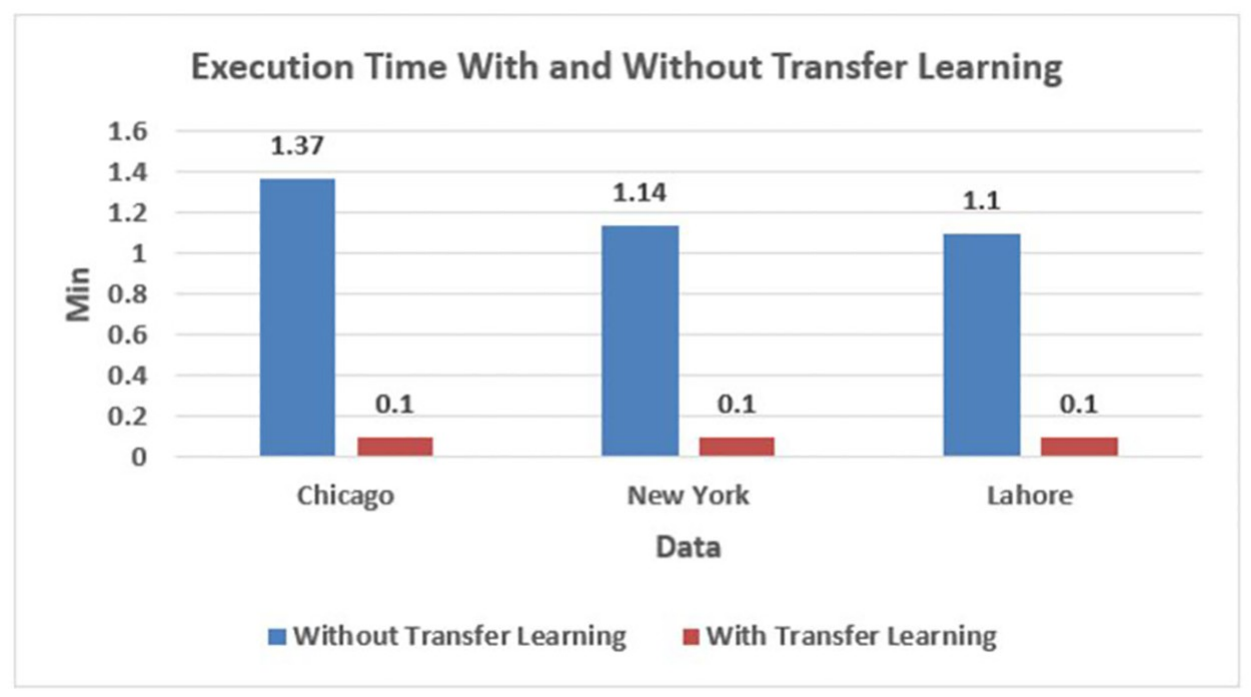
Comparison of datasets over execution time for a month using transfer learning.

Fig 12 compares execution time with transfer learning and without transfer learning used to predict the monthly crime of a district, borough, and town on a bar chart with the dataset name on the X-axis and execution time on the Y-axis.

## 5 Conclusion

Crimes represent a severe danger to human civilization, security, and long-term growth and are expected to be managed. Therefore, law enforcement agencies frequently demand computational forecasts and prediction-based systems that improve crime analytics to improve city safety and security and prevent criminal activity. Furthermore, the availability of spatiotemporal crime data is vital to predicting crimes. Several studies have highlighted the significance of deep learning methods in enhancing crime prediction accuracy. However, sufficient data and resources are required to train a deep learning system. Thus, this study used the transfer learning paradigm and deep learning techniques to predict crime. This study employed and fine-tuned diverse statistical, deep learning, and machine learning algorithms on crime datasets from Chicago, New York, and Lahore. Moreover, EDA also highlights daily, weekly, monthly, yearly, and hourly crime trends.

BiLSTM achieves the maximum performance and low MAE, MAD, and MSE rates among the various algorithms. Therefore, this study exploited BiLSTM under transfer learning to predict monthly crime trends. The proposed approach achieves comparable performance, a low error rate, and less execution time by transferring knowledge from District 1 to District 2, Brooklyn to Manhattan, and from Iqbal town to Nishtar town. The proposed approach is significant for law enforcement agencies in predicting crime with fewer resources and time. In the future, the authors aim to fine-tune the knowledge transfer mechanism at the parameter level to avoid negative transfers. Moreover, cross-region (Lahore to Chicago or New York to Lahore) knowledge transfer will be studied as crime trends in EDA show similar characteristics.
